# Innovative animal component-free surface for the cultivation of human embryonic stem cells

**DOI:** 10.1186/1753-6561-5-S8-P51

**Published:** 2011-11-22

**Authors:** Thomas Stelzer, Tina Marwood, Cindy Neeley

**Affiliations:** 1 Thermo Fisher Scientific Labware and Specialty Plastics, Roskilde, DK-4000, Denmark; 2 Thermo Fisher Scientific Labware and Specialty Plastics, Rochester, NY 14625, USA

## Background

The promise of pluripotent stem cells lies in their ability to form any cell or tissue in the body. However, this promise requires a stable and reproducible method to grow the cells. Current methods rely on feeder cells or extracellular matrix proteins to cover the cultureware growth surface, and either manual selection or enzymatic dissociation in cell passaging and harvesting. This study describes a novel and simple method to grow pluripotent stem cells without the use of feeder cells or extracellular matrix proteins.

## Materials and methods

Human ESC cultivation

Cells: Passage-49 human ESC (H1 line from WiCELL, USA) were maintained in mouse embryonic fibroblast (MEF)-conditioned medium on Nunclon™ Delta™ surface (Thermo Fisher Scientific, USA) coated with a 1:30 dilution of growth-factor reduced Matrigel™ (Becton Dickinson, USA). Cells were dissociated from the surface for passage by treatment with 1 mg/ ml collagenase, and then seeded onto Nunclon Vita™ surface with or without Rho-kinase inhibitor in the medium, as described below.

Cultivation without Rho-kinase inhibition: H1 ESC was grown for 4 passages in MEF-conditioned medium on Nunclon Vita surface. Cells were dissociated from the surface for passage by treatment with 1 mg/ml collagenase. Cells plated on the Nunclon Vita surface took 7 days of culturing before they were ready for passage.

Cultivation with Rho-kinase inhibition: H1 ESC were grown in MEF-conditioned medium supplemented with Rho-kinase inhibitor, Y-27632 (10 µM unless otherwise indicated; Sigma-Aldrich, USA). Cells were dissociated from the surface for passage by treatment with 1 mg/ml collagenase. Cells plated in medium with 10 µm Y-27632 on the Nunclon Vita surface were ready for passage 4 days after plating. Cells were grown for the number of passages as indicated.

Human ESC characterization: Colony presence and morphology were determined using phase-contrast microscopy, and by the naked eye after staining colonies with 0.5% crystal violet.

Pluripotency was determined by the presence of pluripotency markers through the use of qRT-PCR for gene expression, flow cytometry for cell surface marker expression, and immunofluorescence for cell-surface and nuclear proteins.

Karyotypic stability was determined by cytogenetic analysis of 20 G-banded metaphase cells, and by fluorescent in situ hybridization (FISH) on 200 interphase nuclei using probes for the ETV6 BAP (TEL) gene located on chromosome 12 and for chromosome 17 centromere.

Ability to form embryoid bodies was determined by growing ESC in a low-binding plate for 10 days in DMEM/F12 containing 10% FBS.

## Results

Human ESCs can be passaged a few times on the Nunclon Vita surface in MEF-conditioned media without Rho-kinase inhibition before the growth rate spontaneously declines. Such decline in growth rate of human ESC on the Nunclon Vita surface is prevented by supplementing the conditioned medium with Rho-kinase inhibitor Y-27632.

**Figure 1:**
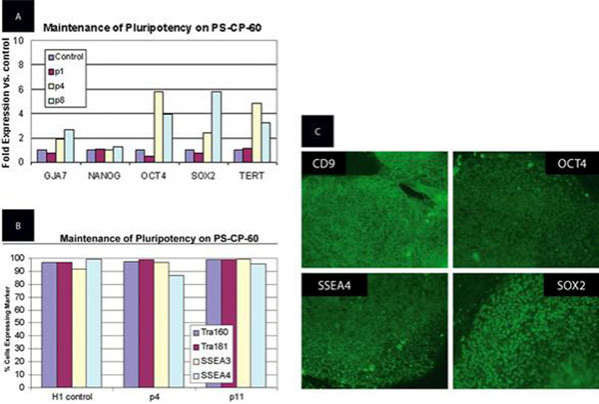
**A.** Expression of pluripotency markers in human ESC as determined by qRT-PCR after 1 passage (p1), 4 passages (p4), and 8 passages (p8) on the Nunclon Vita surface. **B.** Expression of pluripotency markers in human ESC as determined by flow cytometry after 4 passages (p4) and 11 passages (p11) on the Nunclon Vita surface. **C.** Expression of pluripotency markers in human ESC as determined by immunofluorescence staining after 11 passages on the Nunclon Vita surface.

Human ESCs grown on the Nunclon Vita surface in the presence of Y-27632 have normal karyotype, express pluripotency markers (Fig. 1), and can be differentiated into embryoid bodies. Non-enzymatic passaging of human ESCs on Nunclon Vita surface can be accomplished by removing Y-27632 from the culture media 15 to 30 minutes before sub-culturing.

## Conclusions

The Nunclon Vita surface support feeder cell- and extracellular matrix-free attachment, colony formation and growth of human ESC:

• For a few passages in media conditioned by mouse embryonic fibroblasts

• For over 10 passages in media conditioned by mouse embryonic fibroblasts and supplemented with Rho-kinase inhibitor Y-27632. The human ESCs expanded 11 passages on the Nunclon Vita surface maintained normal karyotype, pluripotency, and their ability to differentiate into germ layer cells.

